# Viruses Causing Aseptic Meningitis: A Tertiary Medical Center Experience With a Multiplex PCR Assay

**DOI:** 10.3389/fneur.2020.602267

**Published:** 2020-12-07

**Authors:** Mohammed A. Aldriweesh, Edi A. Shafaay, Saud M. Alwatban, Obeid M. Alkethami, Faisal N. Aljuraisi, Mohammad Bosaeed, Naif Khalaf Alharbi

**Affiliations:** ^1^College of Medicine, King Saud bin Abdulaziz University for Health Sciences, Riyadh, Saudi Arabia; ^2^Division of Infectious Diseases, Department of Medicine, King Abdulaziz Medical City, Riyadh, Saudi Arabia; ^3^Department of Infectious Disease Research, King Abdullah International Medical Research Center, Riyadh, Saudi Arabia

**Keywords:** diagnostic PCR, enterovirus, herpesvirus, CNS infection, Filmarray ME panel, viral meningitis

## Abstract

**Background:** Central nervous system (CNS) infection is associated with high rates of morbidity and mortality, and despite advancements in molecular testing, aseptic meningitis remains challenging to diagnose. Aseptic meningitis cases are often underreported worldwide, which impacts the quality of patient care. Therefore, we aimed to assess the results of BioFire® FilmArray® meningitis/encephalitis (ME) PCR panel, clinical characteristics, and etiologies of aseptic meningitis patients.

**Methods:** From January 2018 to January 2020, all pediatric and adult patients in a large tertiary medical center who underwent lumbar puncture and cerebrospinal fluid (CSF) testing by a ME multiplex PCR panel and who fit the aseptic meningitis definition were retrospectively reviewed.

**Results:** Data were reviewed from 1,607 patients; 240 met the inclusion criteria (54.6% males; 68.8% <4 years of age). The rate of detected viral causes of aseptic meningitis was 40.4%; therefore, 59.6% of the patients remained with unidentified etiology. Among the identified viral meningitis, enterovirus and human herpesvirus 6 (HHV-6) were the most common (25 and 7.9%, respectively). The median length of hospital stay was 6 days, and it was longer in patients with unidentifiable aseptic meningitis (*p* < 0.0001).

**Conclusion:** Aseptic meningitis is common among suspected meningitis patients, but most cases remained of unknown etiology. The most common identified viruses were enterovirus followed by HHV-6, and there is predominance in males and the pediatric age group. These results highlight that further research is needed to identify other etiologies and possible additional viral pathogens for aseptic meningitis in the current diagnostic methods.

## Introduction

Central nervous system (CNS) infections—whether by bacteria, fungi, protozoa, or viruses—are neurological emergencies requiring urgent medical intervention (1–3). CNS infections are diagnosed as meningitis, encephalitis, and meningoencephalitis, depending on the presence of meningeal signs, focal signs, and altered brain functions, respectively (4).

Aseptic meningitis, which is the most common form of meningitis, is confirmed when there is presence of acute onset of meningeal signs and cerebrospinal fluid (CSF) pleocytosis [CSF white blood cells (WBC): ≥5 cells/mm^3^] with negative bacterial and fungal cultures (5–8). Importantly, the viral etiology of the majority of aseptic meningitis cases is unknown (9–11). Generally, the diagnosis of meningitis is clinically challenging; however, polymerase chain reaction (PCR) testing helps distinguish between viral, bacterial, and fungal meningitis (5, 12). Using PCR, several international studies reported more viral than bacterial or fungal meningitis cases (13–15).

Since the introduction of vaccines, the rate of bacterial meningitis has dropped significantly, with nearly complete elimination of some types of viral and bacterial meningitis (16). Before the measles, mumps, and rubella (MMR) vaccine, mumps-related meningitis was the leading cause of viral meningitis (17, 18). Viral meningitis is frequently reported in pediatric populations; however, enteroviruses are responsible for many cases in both pediatric and adult populations (11, 12, 18–23).

In the United Kingdom (UK), a study reported an estimated annual incidence of viral meningitis in adults as 2.73 per 100,000 (19). However, the hospital admission rate of viral meningitis in those aged <15 years was reported as 13.5 per 100,000, with the same rate found in infants (18). In the Arabian Gulf Cooperation Council countries, data are limited; however, a recent study in Qatar found that viral meningitis is the most common form of CNS infection, with enterovirus being the most common cause (21). In the Kingdom of Saudi Arabia (KSA), no studies have yet investigated the etiologies of viral meningitis using PCR multiplex panels.

Therefore, this study aimed to contribute to the literature on aseptic (viral) meningitis in the KSA, more specifically by assessing the most common etiology of aseptic meningitis among pediatric and adult patients.

## Materials and Methods

### Study Design and Settings

This retrospective cohort study was conducted at King Abdulaziz Medical City (KAMC) in Riyadh, Saudi Arabia. KAMC is a tertiary medical center with a bed capacity of 1,501. KAMC also encompasses King Abdullah Specialist Children's Hospital (KASCH), which has a 600-bed capacity. The study was designed to investigate the rates and etiologies of aseptic meningitis, clinical patient characteristics, mortality rate, length of stay, and treatment among patients who underwent lumbar puncture (LP) between January 2018 and January 2020 and who had available results from CSF testing with the BioFire® FilmArray® meningitis/encephalitis (ME) multiplex PCR panel.

### Study Participants

**Figure 1:**
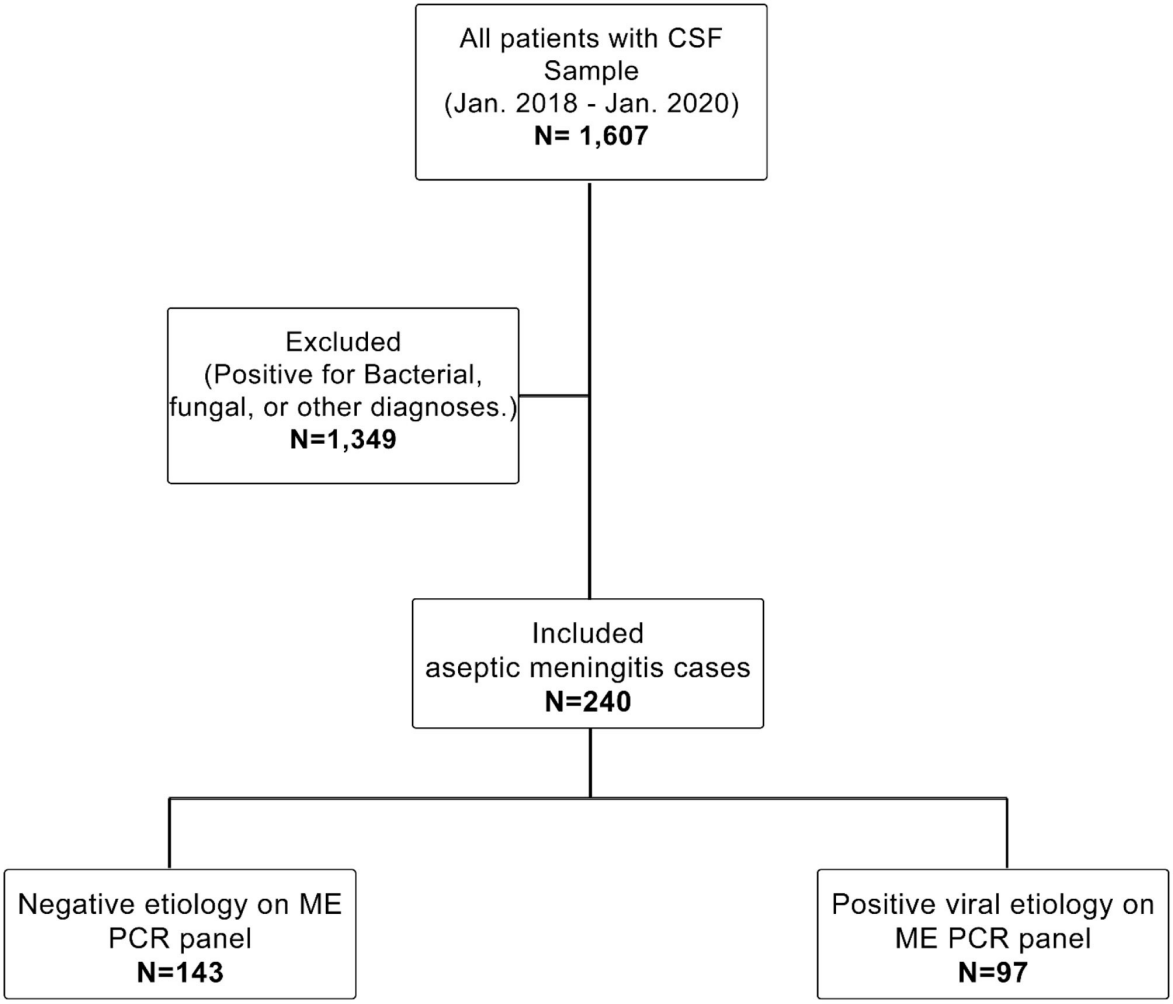
Study cohort. Shows the population inclusion and exclusion criteria. The study reviewed all patients with available CSF samples from Jan. 2018 to Jan. 2020 (*N* = 1,607). We excluded every sample with positive fungal or bacterial culture or other diagnoses (*N* = 1,349). Finally, only patients with aseptic meningitis were included in this study (*N* = 243). The included cases were subdivided based on the ME PCR panel into a positive viral etiology group (*N* = 97) and a negative viral etiology group (*N* = 143).

The patients included had LP and CSF testing by PCR, and they fit the definition for aseptic meningitis developed by Wright et al., namely, the presence of acute onset of meningeal signs and CSF pleocytosis [CSF white blood cells (WBC): ≥5 cells/mm^3^] with negative bacterial and fungal cultures and the exclusion of any patient who presented with a clinical picture of encephalitis such as cranial nerve palsies, paresis or paralysis, altered reflexes, or convulsions (6). Moreover, all ICU patients were reviewed to exclude autoimmune/infectious encephalitis, drug-induced meningitis, and other diagnoses such as vascular diseases, brain tumors, and traumatic brain injuries. Aseptic meningitis patients were divided into two groups: patients with positive viral etiology in the ME multiplex PCR panel and those with negative viral etiology. The sampling technique used was purposive sampling, as shown in Figure 1.

### Data Collection

The electronic medical records of the included patients were reviewed. Signs, symptoms, and temperature (°C) were collected at the first presentation to the emergency department. Travel history, contact history, and comorbidities were collected from consultation notes throughout the hospitalization course. CSF results, including CSF glucose, CSF protein, and CSF WBC, were collected from the laboratory at the first-time test. PCR results were collected from the records.

### Laboratory Diagnostic Procedure

All CSF samples were tested for PCR, cultures, and cell count/differential. For PCR, the BioFire® FilmArray® ME panel, a qualitative multiplex real-time PCR assay, was used. Nucleic acids were automatically extracted and processed in a multiplex PCR assay for enterovirus, herpes simplex virus type 1 and 2 (HSV-1/HSV-2), varicella-zoster virus (VZV), cytomegalovirus (CMV), human herpesvirus 6 (HHV-6), and human parechovirus (HPeV).

### Statistical Analysis

Data were analyzed using Statistical Package for Social Sciences (SPSS) v. 22 (Chicago, IL, USA). Percentages and frequencies were used for categorical variables (e.g., gender). Continuous variables were analyzed as mean, standard deviation, median, and interquartile range (IQR) for non-normally distributed variables. Inferential statistics were applied to compare the demographic and clinical characteristics between positive and negative patients and compare between patients who received antibiotics alone or antiviral-based treatment on the independent *t*-test and chi-square tests for continuous and categorical variables, respectively. A *p* < 0.05 was considered significant.

### Ethical Approval

The study received approval from the Institutional Review Board (IRB) committee (RC19/242/R) at King Abdullah International Medical Research Center (KAIMRC), the Ministry of National Guard Health Affairs.

## Results

Of the 1,607 patient files reviewed, 240 met the inclusion criteria; their demographic and clinical characteristics are presented in Table 1. Among these 240 patients (54.6% male), the rate of positive viral meningitis was 40.4% (*n* = 97), and the rate of aseptic meningitis of unknown etiology was 59.6% (*n* = 143). The majority of patients with viral meningitis were <4 years of age (66%), while 18.6% were 5–17 years of age. The most common symptom in positive viral meningitis cases was fever (72.2%), followed by headache (25.8%) and vomiting (20.6%); few patients (5.2%) presented with neck pain/stiffness, and Kernig's and Brudzinski's signs were rarely reported (2.1 and 2.1%, respectively). Among the 97 patients, 14 (14.4%) were admitted to the intensive care unit (ICU), 9 of which (9.2%) were pediatric cases. None of the ICU cases were diagnosed with encephalitis. Death was reported in three viral meningitis patients, all of whom were immunocompromised patients.

**Table 1:** The demographic and clinical characteristics of aseptic meningitis patients with positive viral etiology^a^.

**Variable**	***N* (%)**
**Gender**	
Male	52 (53.6%)
Female	45 (46.4%)
**Age groups**	
0–4 years	64 (66%)
5–17 years	18 (18.6%)
18–34 years	6 (6.2%)
35–64 years	5 (5.2%)
≥65 years	4 (4.1%)
**Symptoms**	
Fever	70 (72.2%)
Headache	22 (25.8%)
Vomiting	20 (20.6%)
Photophobia	10 (10.3%)
Hypoactivity	13 (13.4%)
Decreased oral intake	10 (10.3%)
Seizure	8 (8.2%)
Altered mental status	6 (6.2%)
**Signs**	
Neck pain/stiffness	5 (5.2%)
Kernig's sign	2 (2.1%)
Brudzinski's sign	2 (2.1%)
**Temperature (°C) (mean ± SD)**	37.7 ± 0.8
**Median Length of Stay (IQR)**	2 (2–4)
**Sepsis**	3 (3.1%)
**Intensive care unit (ICU) admissions**	14 (14.4%)
**Death**	3 (3%)
**CSF characteristics**	
Median WBC (cells/mm^3^) (IQR)	15 (1–84.5)
Median protein (g/L) (IQR)	0.5 (0.32–0.81)
Median glucose (mmol/L) (IQR)	3 (2.7–3.4)

a *Patients with positive ME multiplex panel (n = 97)*.

The viruses we detected in aseptic meningitis cases are presented according to gender and age groups (Table 2). The most prevalent virus was enterovirus (25%; *n* = 60), followed by HHV-6 (7.9%; *n* = 19), and VZV (3.8%; *n* = 9). Enterovirus was common in the pediatric age group, especially in those younger than 4 years of age. Enterovirus was only reported in four cases in adults, an age group encompassing 18–34 year-olds. HSV-2 was detected in two female cases in the 34–64 year age group. However, in the majority of patients (59.6%; *n* = 143), the causative pathogen was not identified.

**Table 2:** The etiology of identified viral meningitis by gender and age groups.

**Virus**	**No. of detected**	**% of positive samples**	**Gender**		**No. of positive detections by age group (years)**				
			**Male**	**Female**	**0–4**	**5–17**	**18–34**	**35–64**	**≥65**
CMV	3	1.3%	2	1	2	0	1	0	0
Enterovirus	60	25%	32	28	41	15	4	0	0
HSV2	2	0.8%	0	2	0	0	0	2	0
HHV-6	19	7.9%	8	11	16	2	0	1	0
HPeV	4	1.7%	3	1	4	0	0	0	0
VZV	9	3.8%	7	2	1	1	2	2	4
Unknown^a^	0	0%	79	64	101	15	15	10	2

*CMV, cytomegalovirus; HSV-2, herpes simplex virus; HHV-6, human herpesvirus 6; HPeV, human parechovirus; VZV, varicella-zoster virus*.

a *The patient had negative results on the ME multiplex panel*.

Demographic and clinical characteristics were compared between patients with positive and negative viral multiplex PCR panel results (Table 3). No statistically significant differences were found between the two groups in terms of gender and age group. However, fever and temperature differed significantly between the two groups (*p* < 0.0001). The temperature was higher in the positive group (37.8 ± 0.88 vs. 37.4 ± 0.9°C; *p* < 0.0001). Neck pain/stiffness was observed in 11.2% (*n* = 16) of the negative group and in 5.2% (*n* = 5) of the positive group. The length of stay was longer in the PCR-negative group (*p* < 0.0001), with a median duration of 2 days in the positive group and 6 days in the negative group. Sepsis was reported in 6.3% (*n* = 9) of the negative group and in 3.1% (*n* = 3) of the positive group.

**Table 3:** The demographic and clinical characteristics of aseptic meningitis patients with positive and negative viral multiplex PCR data.

**Variable**	**Positive** ***N* (%)** **(*N* = 97)**	**Negative** ***N* (%)** **(*N* = 143)**	***p***
**No. of patients**	97 (40.4%)	143 (59.6%)	
**Gender**			
Male	52 (53.6%)	79 (55.2%)	0.8
Female	45 (46.4%)	64 (44.8%)	
**Age groups**			
0–4 years	64 (66%)	101 (70.6%)	0.18
5–17 years	18 (18.6%)	15 (10.5%)	
18–34 years	6 (6.2%)	15 (10.5%)	
35–64 years	5 (5.2%)	10 (7%)	
≥65 years	4 (4.1%)	2 (1.4%)	
**Symptoms**			
Fever	70 (72.2%)	73 (51%)	**<0.0001**
Headache	25 (25.8%)	27 (18.9%)	0.2
Vomiting	20 (20.6%)	31 (21.7%)	0.87
Photophobia	10 (10.3%)	10 (7%)	0.47
Hypoactivity	13 (13.4%)	17 (11.9%)	0.84
Decreased oral intake	10 (10.3%)	17 (11.9%)	0.83
Seizure	8 (8.2%)	10 (7%)	0.8
Altered mental status	6 (6.2%)	0	
**Signs**			
Neck pain/stiffness	5 (5.2%)	16 (11.2%)	0.16
Kernig's sign	2 (2.1%)	5 (3.5%)	0.7
Brudzinski's sign	2 (2.1%)	5 (3.5%)	0.7
**Temperature (°C)** (mean ± SD)	37.8 ± 0.8	37.4 ± 0.9	**<0.0001**
**Median length of stay** (IQR)	2 (2–4)	6 (2–17)	**<0.0001**
**Sepsis**	3 (3.1%)	9 (6.3%)	0.37
**Intensive care unit (ICU) admissions**	14 (14.4%)	40 (28%)	**0.01**
**Death**	3 (3.1%)	0	
**CSF characteristics**			
Median WBC (cells/mm^3^) (IQR)	15 (1–84.5)	13 (8–44)	**<0.0001**
Median protein (g/L) (IQR)	0.5 (0.32–0.81)	0.82 (0.47–1.4)	0.7
Median glucose (mmol/L) (IQR)	3 (2.7–3.4)	3.1 (2.7–3.8)	**0.02**

*The bold values showed statistical significance (A p < 0.05 was considered significant)*.

*P-value indicates statistical significance as obtained from chi-square for categorical variables and independent-samples t-test for continuous variables*.

Of the positive group, 14.4% (*n* = 14) were admitted to the ICU, relative to 28% (*n* = 40) of the negative group. Death was reported in only three cases in the positive group, all of which were attributed to VZV infection with a known history of comorbidities and co-infection. The first case had VZV and co-infection with *Candida albicans* in urine, with a medical history that includes hypertension, diabetes mellitus, ischemic heart disease, chronic kidney disease, and immune thrombocytopenic purpura (ITP). The second case had VZV with a significant medical history of systemic lupus erythematosus (SLE), CNS lymphoma, and co-infection with blood CMV. The third patient had diffuse large B-cell lymphoma, ovarian cancer, and adrenal insufficiency (on long-term corticosteroids); was dual positive for CSF CMV and VZV; and co-infected with *Proteus mirabilis* and *Acinetobacter bumannii*. With respect to CSF characteristics, the median CSF WBC was 15 cells/mm^3^ (positive group) vs. 13 cells/mm^3^ (negative group); *p* < 0.0001. Similarly, CSF glucose levels were higher in the negative group (3.0 vs. 3.1 mmol/L; *p* = 0.02). Regarding CSF protein, the median was 0.82 g/L in the negative group and 0.5 g/L in the positive group.

**Figure 2:**
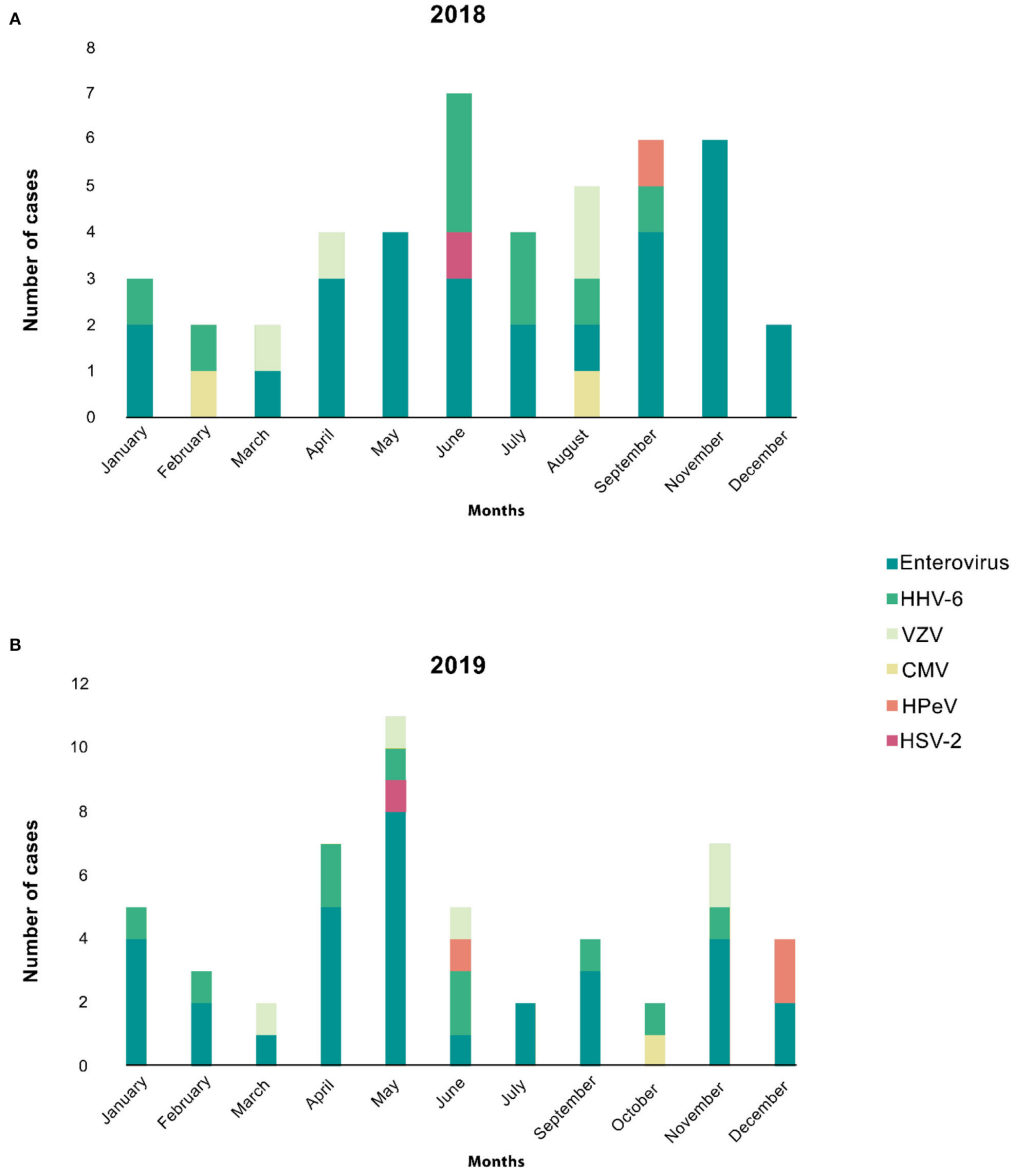
The monthly distribution of aseptic meningitis cases is stratified by different viruses. **(A)** Shows the trend and seasonality of viruses between Jan 2018 and Dec 2018; no identified viruses were reported in October. The figure shows a trend of enteroviral meningitis in November, May, followed by September. **(B)** Depicts the trend and seasonality of viruses between Jan 2019 and Dec 2019; we reported no August cases. The figure shows a trend of enteroviral meningitis in May, April, followed by November. However, other viruses showed neither relation to seasonality nor any trends in both years.

The monthly distribution of aseptic meningitis with positive viral etiologies from January 2018 to January 2020 is shown in Figure 2, highlighting the seasonality of positive viral meningitis. The viruses detected in our study do not appear to have a seasonal occurrence. Figure 2A shows the trend and seasonality of viruses between January 2018 and December 2018; no identified viruses were reported in October. The figure shows a trend of enteroviral meningitis in November and May followed by September. Figure 2B depicts the trend and seasonality of viruses between January 2019 and December 2019; we reported no August cases. Enteroviral meningitis was noted to have a trend in May and April followed by November. We reported only one case with an unidentifiable virus in January 2020. No specific correlations were found between any other virus and a particular month or season in both years.

Lastly, this study investigated treatment effectiveness. Table 4 describes the difference between treatments in the two groups. No statistical significance was found between the two groups with respect to sepsis, ICU admission, and length of hospital stay.

**Table 4:** The effect of antibiotics alone vs. antibiotics and antiviral treatment on the clinical course^a^.

**Variable**	**Antibiotics alone** **(*N* = 186)** ***N* (%)**	**Antibiotics and antiviral** **(*N* = 62)** ***N* (%)**	***p***
Sepsis	10 (5.5%)	2 (4%)	0.66
ICU admission	46 (25.4%)	7 (14%)	0.12
Median length of stay (IQR)	3 (2–11)	3 (1–13)	0.89

a *Including patients from positive and negative groups, with available treatment data (n = 243)*.

*P-value indicates statistical significance as obtained from chi-square for categorical variables and independent-samples t-test for continuous variables*.

*(A p < 0.05 was considered significant)*.

## Discussion

The present study was designed to assess the utility of ME PCR panel testing in detecting the viral etiologies of aseptic meningitis in pediatric and adult patients in a single large medical center in Saudi Arabia. The rate of detected viruses among aseptic meningitis patients was found to be 40.4%. The incidence rate of aseptic meningitis in the Gulf region ranges from 7.2 to 38% (21, 24, 25). In this cohort, the most common viral cause of aseptic meningitis was enterovirus (25%), followed by HHV-6 (7.9%); these data corroborate the findings of earlier reports (21–23).

The diagnostic tests currently used to detect microorganisms that cause CNS infections are routine culture and advanced technologies such as multiplex PCR assay and metagenomic next-generation sequencing (mNGS) (26–28). Despite advances in molecular technologies allowing for rapid and accurate diagnosis, aseptic meningitis remained challenging to diagnose, given that we were unable to identify the causative pathogen in 59.6% of our sample. Meningitis of unknown cause was reported to be 62% in Qatar, 81.5% in the USA, and 42% in the UK (11, 19, 21). Unknown causes were probably due to the limitations of laboratory testing, such as the limitation of ME PCR panel testing for a few types of viruses. This indicates worsening cases in the absence of a diagnosis that correctly identifies the underlying cause. Some clinicians would send samples for further outsourced testing for specific meningitis-causing viruses, such as Epstein–Barr virus (EBV), BK, and JC (29–31).

In this cohort, we were unable to identify any EBV cases since it is not among the ME multiplex viruses. A study used MassTag (mNGS) and compared it to conventional qPCR to investigate the causes of bacterial and viral CNS infections, and the study found EBV to be the most common cause of viral CNS infections (32). Given the limited list of viruses in the current standard PCR panels for meningitis, as well as the uneasiness of collecting further CSF samples for additional testing, there is a clear need for more inclusive diagnostic tools (33); some reports have already called for the inclusion of BK and JC to be in the routine standard testing such as ME multiplex panel (34).

This study, and others, highlights the importance of developing or broadening the current molecular tests to identify more viral agents. A new emerging technique that might be very broad yet highly accurate at detection is the mNGS, which has been shown to detect more neuroinvasive viruses accurately (32, 35). In fact, mNGS was able to identify 16 pathogens that could not otherwise be detected by routine methods (35), attesting to its robustness and sensitivity. Another study used mNGS, VirScan, and serology and found a high rate of enterovirus detection (up to 92%) relative to routine qRT-PCR and mNGS (36). However, with advanced techniques such as mNGS, costs are significantly higher than those of routine PCR tests. Nevertheless, most of the aseptic meningitis cases have a manageable course of the disease that does not necessarily require further analysis. This is also important in the context of cost-effectiveness when evaluating clinical management costs against diagnostic costs and utility.

Regarding the clinical features in this study, a predominance of aseptic meningitis was noted in males (53.6%), which supports previous research that showed higher viral CNS infections in males (21). This can be explained by the male gender predominance (59.2%) in Riyadh, the capital city of KSA (37). In the context of age group and clinical presentation, our study is quite similar to a regional study in Qatar, wherein the most affected age group was pediatrics (0–4 years old) and the three most common symptoms were fever, headache, and vomiting (21).

In the current study, ICU admission was reported in 54 cases; most of them were in the negative group (28%; *n* = 40). Some of the ICU cases were admitted due to sepsis, immunosuppression, or requiring a 24-h observation by the ICU team. A previous study reported a similar rate of ICU admissions in aseptic meningitis patients (38).

The mortality rate of aseptic meningitis in our study was 1.1%. All deaths occurred in immunocompromised patients, who are typically known to be infected with fatal opportunistic pathogens; in these cases, fatal sepsis had developed. Of note, VZV was reported in all of the three fatal cases, while two of the cases also had co-infection with CMV; these cases also had a significant medical history, including autoimmune diseases such as ITP and SLE. Other studies reported VZV as the most common viral infection among ITP patients (39). SLE patients are on lifetime immunosuppressants and at high risk for viral infections, especially VZV (40). Moreover, the third case was severely immunocompromised, highlighting the importance of aseptic meningitis in this group of patients.

The study has potential limitations that include missing cases due to the retrospective nature of the study, limited generalizability of any data trends or conclusions, and the lack of a historic control group with data prior to the implementation of the ME multiplex PCR panel. However, this study also has strengths, namely, it used a standardized and broadly used assay, and it is the first report of its kind locally.

## Conclusion

In this paper, 40.4% of the aseptic meningitis cases were caused by identifiable viruses using the ME PCR panel; most of these cases were males, in the pediatric age group, and diagnosed with enterovirus. A low mortality rate of 1.1% is reported in this study, all of which occurred in immunocompromised patients. Although there are advances in detecting viruses by molecular testing, etiologies of aseptic meningitis remain underdiagnosed. This suggests the need for broadening the existing ME multiplex PCR panels or increasing the use of emerging advanced technologies such as mNGS. Overall, in the absence of comprehensive and widely available molecular testing, better clinical management for aseptic meningitis is warranted, and further work is highly recommended to expand our knowledge of neuroinvasive viruses.

## Data Availability Statement

The raw data supporting the conclusions of this article will be made available by the authors, without undue reservation.

## Author Contributions

MA, NA, and ES contributed to the design and implementation of the research and the writing of the manuscript. MA analyzed the data and wrote the methodology. ES, SA, OA, and FA collected the data and co-wrote the manuscript. NA and MB reviewed and edited the final manuscript. NA supervised the project. All authors agreed on the final manuscript.

## Conflict of Interest

The authors declare that the research was conducted in the absence of any commercial or financial relationships that could be construed as a potential conflict of interest.

## References

[1] Fitch MT van de Beek D. Emergency diagnosis and treatment of adult meningitis. Lancet Infect Dis. (2007) 7:191–200. 10.1016/S1473-3099(07)70050-6 17317600

[2] Fitch MT Abrahamian FM Moran GJ Talan DA. Emergency department management of meningitis and encephalitis. Infect Dis Clin North Am. (2008) 22:33–52. 10.1016/j.idc.2007.10.001 18295682

[3] Richard GC Lepe M Meningitis in children: diagnosis and treatment for the emergency clinician. Clin Pediatr Emerg Med. (2013) 14:146–56. 10.1016/j.cpem.2013.04.008

[4] Calleri G Libanore V Corcione S De Rosa FG Caramello P. A retrospective study of viral central nervous system infections: relationship amongst aetiology, clinical course and outcome. Infection. (2017) 45:227–31. 10.1007/s15010-017-0993-4 28236249

[5] Case definitions for infectious conditions under public health surveillance Centers for disease control and prevention. MMWR Recomm Rep. (1997) 46:1–55. 9148133

[6] Wright WF Pinto CN Palisoc K Baghli S. Viral (aseptic) meningitis: a review. J Neurol Sci. (2019) 398:176–83. 10.1016/j.jns.2019.01.050 30731305

[7] McGill F Griffiths MJ Solomon T Viral meningitis. Curr Opin Infect Dis. (2017) 30:248–56. 10.1097/QCO.0000000000000355 28118219

[8] Logan SAE MacMahon E. Viral meningitis. BMJ. (2008) 336:36–40. 10.1136/bmj.39409.673657.AE 18174598 PMC2174764

[9] Mount HR Boyle SD. Aseptic and bacterial meningitis: evaluation, treatment, and prevention. Am Fam Phys. (2017) 96:314–22. 28925647

[10] Jarrin I Sellier P Lopes A Morgand M Makovec T Delcey V. Etiologies and management of aseptic meningitis in patients admitted to an internal medicine department. Medicine. (2016) 95:e2372. 10.1097/MD.0000000000002372 26765411 PMC4718237

[11] Shukla B Aguilera EA Salazar L Wootton SH Kaewpoowat Q Hasbun R. Aseptic meningitis in adults and children: diagnostic and management challenges. J Clin Virol. (2017) 94:110–4. 10.1016/j.jcv.2017.07.016 28806629 PMC5581214

[12] Kadambari S Okike I Ribeiro S Ramsay ME Heath PT Sharland M. Seven-fold increase in viral meningo-encephalitis reports in England and Wales during 2004–2013. J Infect. (2014) 69:326–32. 10.1016/j.jinf.2014.05.012 24887614

[13] Boudet A Pantel A Carles MJ Boclé H Charachon S Enault C. A review of a 13-month period of filmarray meningitis/encephalitis panel implementation as a first-line diagnosis tool at a university hospital. PLoS ONE. (2019) 14:e0223887. 10.1371/journal.pone.0223887 31647847 PMC6812749

[14] Radmard S Reid S Ciryam P Boubour A Ho N Zucker J. Clinical utilization of the filmarray meningitis/encephalitis (ME) multiplex polymerase chain reaction (PCR) assay. Front Neurol. (2019) 10:281. 10.3389/fneur.2019.00281 30972012 PMC6443843

[15] Naccache SN Lustestica M Fahit M Mestas J Barda JD. One year in the life of a rapid syndromic panel for meningitis/encephalitis: a pediatric tertiary care facility's experience. J Clin Microbiol. (2018) 56:1–11. 10.1128/JCM.01940-17 29540454 PMC5925728

[16] McIntyre PB O'Brien KL Greenwood B Van De Beek D. Effect of vaccines on bacterial meningitis worldwide. Lancet. (2012) 380:1703–11. 10.1016/S0140-6736(12)61187-8 23141619

[17] Galbraith NS Pusey J Young SJ Crombie DL Sparks JP. Mumps surveillance in England and Wales 1962–81. Lancet. (1984) 323:91–4. 10.1016/S0140-6736(84)90015-1 6140434

[18] Martin NG Iro MA Sadarangani M Goldacre R Pollard AJ Goldacre MJ. Hospital admissions for viral meningitis in children in England over five decades: a population-based observational study. Lancet Infect Dis. (2016) 16:1279–87. 10.1016/S1473-3099(16)30201-8 27527749

[19] McGill F Griffiths MJ Bonnett LJ Geretti AM Michael BD Beeching NJ. Incidence, aetiology, and sequelae of viral meningitis in UK adults: a multicentre prospective observational cohort study. Lancet Infect Dis. (2018) 18:992–1003. 10.1016/S1473-3099(18)30245-7 30153934 PMC6105576

[20] Ai J Xie Z Liu G Chen Z Yang Y Li Y. Etiology and prognosis of acute viral encephalitis and meningitis in Chinese children: a multicentre prospective study. BMC Infect Dis. (2017) 17:494. 10.1186/s12879-017-2572-9 28705180 PMC5513334

[21] Ben Abid F Abukhattab M Ghazouani H Khalil O Gohar A Al Soub H. Epidemiology and clinical outcomes of viral central nervous system infections. Int J Infect Dis. (2018) 73:85–90. 10.1016/j.ijid.2018.06.008 29913285

[22] Hasbun R Wootton SH Rosenthal N Balada-Llasat JM Chung J Duff S. Epidemiology of meningitis and encephalitis in infants and children in the United States, 2011–2014. Pediatr Infect Dis J. (2019) 38:37–41. 10.1097/INF.0000000000002081 30531527

[23] Hudson JA Broad J Martin NG Sadarangani M Galal U Kelly DF. Outcomes beyond hospital discharge in infants and children with viral meningitis: a systematic review. Rev Med Virol. (2019) 30:e2083 10.1002/rmv.2083 31524309

[24] Dash N Ameen AS Sheek-Hussein MM Smego RA. Epidemiology of meningitis in Al-Ain, United arab emirates, 2000–2005. Int J Infect Dis. (2007) 11:309–12. 10.1016/j.ijid.2006.05.009 16950640

[25] Dash N Al Khusaiby S Behlim T Mohammadi A Mohammadi E Al Awaidy S. Epidemiology of meningitis in Oman, 2000-2005. East Mediterr Health J. (2009) 15:1358–64. 20218125

[26] Leber AL Everhart K Balada-Llasat JM Cullison J Daly J Holt S. Multicenter evaluation of biofire filmarray meningitis/encephalitis panel for detection of bacteria, viruses, and yeast in cerebrospinal fluid specimens. J Clin Microbiol. (2016) 54:2251–61. 10.1128/JCM.00730-16 27335149 PMC5005480

[27] Wilson M Tyler KL. Emerging diagnostic and therapeutic tools for central nervous system infections. JAMA Neurol. (2016) 73:1389–90. 10.1001/jamaneurol.2016.3617 27695862 PMC5154841

[28] Tyler KL. What's next (generation) for the diagnosis of chronic meningitis? JAMA Neurol. (2018) 75:915–7. 10.1001/jamaneurol.2018.0473 29710076

[29] Tan CS Koralnik IJ. Progressive multifocal leukoencephalopathy and other disorders caused by JC virus: clinical features and pathogenesis. Lancet Neurol. (2010) 9:425–37. 10.1016/S1474-4422(10)70040-5 20298966 PMC2880524

[30] Behzad-Behbahani A Klapper PE Vallely PJ Cleator GM Bonington A BKV-DNA and JCV-DNA in CSF of patients with suspected meningitis or encephalitis. Infection. (2003) 31:374–8. 10.1007/s15010-003-3078-5 14735377

[31] Bogdanovic G Priftakis P Hammarin A-L Söderström M Samuelson A Lewensohn-Fuchs I Detection of JC virus in cerebrospinal fluid (CSF) samples from patients with progressive multifocal leukoencephalopathy but not in CSF samples from patients with herpes simplex encephalitis, enteroviral meningitis, or multiple sclerosis. J Clin Microbiol. (1998) 36:1137–8. 10.1128/JCM.36.4.1137-1138.1998 9542955 PMC104707

[32] Hasan MR Sundararaju S Tang P Tsui KM Lopez AP Janahi M. A metagenomics-based diagnostic approach for central nervous system infections in hospital acute care setting. Sci Rep. (2020) 10:11194. 10.1038/s41598-020-68159-z 32641704 PMC7343800

[33] Nath A. Neuroinfectious diseases: a crisis in neurology and a call for action. JAMA Neurol. (2015) 72:143–4. 10.1001/jamaneurol.2014.3442 25485794 PMC5267936

[34] Hsu CC Tokarz R Briese T Tsai HC Quan PL Ian Lipkin W. Use of staged molecular analysis to determine causes of unexplained central nervous system infections. Emerg Infect Dis. (2013) 19:1470–7. 10.3201/eid1909.130474 23965845 PMC3810931

[35] Wilson MR Sample HA Zorn KC Arevalo S Yu G Neuhaus J. Clinical metagenomic sequencing for diagnosis of meningitis and encephalitis. N Engl J Med. (2019) 380:2327–40. 10.1056/NEJMoa1803396 31189036 PMC6764751

[36] Leon KE Schubert RD Casas-Alba D Hawes IA Ramachandran PS Ramesh A. Genomic and serologic characterization of enterovirus A71 brainstem encephalitis. Neurol Neuroimmunol Neuroinflamm. (2020) 7:e703. 10.1212/NXI.0000000000000703 32139440 PMC7136061

[37] The General Authority for Statistics (GASTAT) is the official source of statistical data in the Kingdom of Saudi Arabia Retrieved from: https://www.stats.gov.sa/en/43

[38] Debray A Nathanson S Moulin F Salomon J Davido B. Eosinopenia as a marker of diagnosis and prognostic to distinguish bacterial from aseptic meningitis in pediatrics. Eur J Clin Microbiol Infect Dis. (2019) 38:1821–7. 10.1007/s10096-019-03614-y 31230204

[39] Takeoka Y Hino M Oiso N Nishi S Koh KR Yamane T. Virus-associated hemophagocytic syndrome due to rubella virus and varicella-zoster virus dual infection in patient with adult idiopathic thrombocytopenic purpura. Ann Hematol. (2001) 80:361–4. 10.1007/s002770000282 11475151

[40] Danza A Ruiz-Irastorza G. Infection risk in systemic lupus erythematosus patients: susceptibility factors and preventive strategies. Lupus. (2013) 22:1286–94. 10.1177/0961203313493032 24098001

